# A Personal Perspective on Psychology of Aesthetics and the Arts: Ecologically Valid, Interdisciplinary, and Diverse Methodologies

**DOI:** 10.1080/10400419.2023.2269339

**Published:** 2023-11-07

**Authors:** Eva Specker

**Affiliations:** University of Vienna

**Keywords:** empirical aesthetics, psychology of art, everyday aesthetics, methodology, ecological validity

## Abstract

In this invited paper, my aim is to introduce the reader to my body of work by outlining where I think empirical aesthetics, and specifically the study of art, is moving or should be moving toward. I will introduce two main foci of my work: 1) studying art/aesthetics outside of the laboratory and in their “natural habitat,” i.e. doing ecologically valid studies (most commonly in the museum), and 2) methodological aspects of studying art/aesthetic experience in a broad sense: including theory, measurement, and analysis. As future directions, I see a shift toward investigating potential outcomes, as well as ensuring a stronger connection between theory and methodology by incorporating interdisciplinary approaches as well as using more advanced statistical modeling. My aim is to show not just what I have done in the past, but also how this shaped the work that I am currently doing as well as the direction that I see my work developing in and that I believe should be pursued, not just by me but by many others. I chose this format in order to be able to show how I think my work contributes to these developments and can, hopefully, also keep contributing in the future.

As this paper provides a personal perspective (or even journey), it seems fitting to start with a personal start: my discovery of the existence of empirical aesthetics/psychology of art as a field of science. It all started purely by chance: a brief mention Masuda, Gonzalez, Kwan, and Nisbett (2008) in my cross-cultural psychology book. I suddenly realized it was possible to combine my two passions of art and psychology, decided to write my bachelors thesis on the topic and it all snowballed from there. In this paper, I aim to introduce my scientific work that has followed from this initial spark as well as outlining future directions, not just for me specifically but also for the field as whole.

## Ecologically valid studies

My interest in ecologically valid studies generally, and museum studies specifically, started during my master’s program, I believed – and, in fact, still do believe – that the goal is (or should be) to understand how people interact with art in *real life*. Experimental lab work is a necessary and extremely powerful tool to understand people (and their interactions with art), but we always need to assess if these findings translate to the world outside the laboratory.

Based on this premise, I ran a study looking at experiences of awe during art viewing in a museum in Amsterdam to support a line of existing lab studies (Van Elk et al. 2016) and a study comparing art experience in the lab with the museum in New York (Specker, Tinio, & van Elk, 2017) to directly target the role of context/ecological validity.

This latter approach is rather typical of past museum studies – and here I specifically mean the studies done from a psychological perspective – as these generally focus on the impacts of physical environments where people interact with artworks, e.g., seeing the differences in aesthetic experiences between museum exhibition and lab or focus on having an ecologically valid context.

However, little work actually looks at the context or manipulation within museum environments and focuses on how different aspects of this context influence the psychological processing of art. One such difference is that in museum exhibitions artworks are generally placed within a curatorial context or narrative (i.e., a semantic context, for example, they are placed within an artistic movement or the biography of the artist). In contrast, lab studies generally use random presentation of artworks (and for good reason!). I looked at this aspect more specifically in Specker et al. (2020) where we showed that if the curatorial narrative implicitly (i.e., only by visual means = artwork placement among other artworks) communicated deviance, the artist was considered more influential.

A similar point that could be made here is that in this work (Specker et al., 2020) we consider more the experience of the viewer with the exhibition as a whole, similar to Pelowski, Cotter, Specker, et al. (n.d., discussed below) and Reitstätter et al. (2020), who compared the art experience before and after a curatorial rearrangement (and thus at a shift of the entire curatorial context rather than focusing only on narrative). Recently, other work in this direction, specifically Rodriguez et al. (2021), has, in my opinion, very nicely shown the potential of such holistic approaches to understanding art engagement.

Towards the end of my PhD, I then started to focus on one specific aspect that differentiates the museum from the lab: engagement with genuine artworks^1^ (i.e., rather than reproductions) while also looking at context (Grüner, Specker, & Leder, 2019). Surprisingly, we did not find a genuineness effect. As looking at a reproduction on a computer screen seems – at least to me – very different from looking at e.g., an oil painting, I was puzzled and wanted to know more.

This led to a series of studies starting with a meta-analysis (Specker, Fekete, Trupp, & Leder, 2021) that surprisingly also did not show a clear genuineness effect. That is, though we found a small meta-analytic effect (g = 0.32[0.16, 0.47]), most studies (*N* = 8 out of 11) included a context confound. By this, I mean that these studies compared the experience with genuine artworks in the museum, to the experience with reproductions in the lab. This makes it unclear if the effect found is due to the context or due to genuineness. If only looking at studies that exclude this confound, our meta-analysis did *not* find an effect of genuineness.

I then followed-up on this meta-analysis by testing two explanations for the potential lack of this effect: the facsimile accommodation hypothesis (Specker & Leder, 2022) and the anchoring effect (Specker, Arató, & Leder, 2023). *The facsimile accommodation* hypothesis (Locher, Smith, & Smith, 1999, 2001) assumes that we know that we are not looking at “the real thing” and thus, we accommodate for that in our judgments, basically “correcting” this error. In Specker and Leder (2022) we could not find evidence for this explanation. *The anchoring effect* relates to the fact that people make relative judgments, and thus that if a viewer sees only reproductions, they judge these in relation to each other and *not* in relation to the genuine pieces that the viewer does not see (at the time of judging, Specker, Arató, & Leder, 2023). Again, we did not find an effect.

Time to give up? I think not. The work in this direction has convinced me of the necessity of broadening the scope of dependent variables that we look at. Already more than 10 years ago, Silvia (2009) discussed that most empirical aesthetics research focuses on what I would call the “usual suspects,” i.e., liking, pleasure, positive/negative, to which I would add interest and, to a lesser extent, understanding. These have also been the type of aesthetic experiences that research on genuineness has (almost) exclusively looked at. Therefore, for me, this is a clear next step for work on genuineness—i.e. is there a genuineness effect when we look at different dependent variables? Furthermore, I believe this broadening of the scope fits with a current trend in the literature to focus on the outcomes of aesthetic experience.

### Moving to understanding potential outcomes of aesthetic experience

First off, the line between the aesthetic experience itself and the outcome of an aesthetic (or specifically art experience) is not always very clear. A good example is recent interest in the study of empathy and the arts. For example, it has been shown that those who engage with the arts – in a broad sense, i.e. including multiple artistic disciplines and subfields – more frequently are also more empathetic and prosocial (Kou, Konrath, & Goldstein, 2020; Mangione et al., 2018). In this case, empathy is theoretically conceptualized as an outcome: the underlying idea is that art engagement (over time) leads to people becoming more empathic or prosocial, i.e. an increase in trait empathy – however, this idea has not been empirically tested yet.

In contrast, with colleagues, I have looked at state empathy (focusing only on visual art). Specifically, in Pelowski et al. (2020), we assessed the emotional experience of (student) artists while creating artworks and of viewers while looking at these artworks. We found that artists and viewers spontaneously shared emotions (i.e., affective empathy), which viewers felt emotions that artists wanted/intended them to feel more than not intended emotions, and that viewers could guess which emotions the artist wanted them to feel (i.e., cognitive empathy). Recently, we have been able to replicate this work at the Venice Biennale (Pelowski et al., 2022). In this case, the experience of (both cognitive and affective) empathy seems more part of the art experience, rather than an outcome.

Are these two different cases of art and empathy related? In other words: Does experiencing empathy while looking at art also make you more empathic once you leave the exhibition? We started to test this in a recent paper (Pelowski, Cotter, Specker, et al., n.d.), where we, in our second study, used daily-diary methods to assess our participants in daily life for 2 weeks. During this time, they visited an exhibition in the Dom Museum that was focused on power imbalances and acceptance for refugees. In short, we found that there seems to be an effect on empathy, but that this is only short-lived – i.e., people report to be more empathic only on the day of the museum visit and this effect disappears after.

Thus, it seems that a one-off art experience may only have a small and short-lived effect on empathy, but what about repeated engagement/experiences? What if we do not look at a single visit but at the accumulated effect of multiple visits? This would, in my opinion, be crucial to understand. My reasoning here is in line with, e.g., Hartung (2022). She describes the research on narratives and empathy – which has struggled to find consistent effects for single engagements but has found consistent effects of lifetime engagements.

This is a relevant question, not just when we are interested in empathy. Recent work has found similar benefits of the arts – again, in a broad sense including multiple arts disciplines – in terms of mental as well as physical health (Fancourt & Finn, 2019). Here too, the question of one-off vs. repeated engagements is relevant. As Howlin (2022) writes: “as with many other health habits, a ‘little and often’ approach can often lead to the greatest long-term benefits” (p.420). In other words, rather than having one amazingly transformative experience with art, the key may lie in repeated engagement with the arts.

Nonetheless, a crucial aspect is to understand the direction of the effect. To stick with the empathy example: Is it just that empathic people engage more with art? Or can it also be the other way around – that art can make you more empathic?

And it is this crucial point where most work in these directions has been limited, this applies tothe majority of the work included in the scoping review (Fancourt & Finn, 2019) but also the work reviewed by Hartung (2022) as well as the studies discussed above (Pelowski, Specker, et al., n.d.; Kou, Konrath, & Goldstein, 2020; Mangione et al., 2018). Directional (or causal) inference generally relies on the time-sequence of events—e.g., people first visited the museum, then an increase of empathy occurred, or in the case of Kou, Konrath, and Goldstein (2020), art engagement in 2007 predicted pro-social behavior in 2011. Nonetheless, this remains correlational evidence. As such, there can be a confounding variable that may cause both art engagement and pro-social behavior (e.g. empathy), rather than that art engagement leads to pro-social behavior. In addition, this work generally lacks a control group who engage with a non-art or a non-aesthetic experience, making it unclear if potential effects are due to the “art/aesthetic” aspect of the engagement or due to other factors. I refer the reader here to Clift, Phillips, and Pritchard (2021) for a detailed discussion of these issues specific to the field of arts and health.

These limitations are likely enhanced by the fact that there is currently very little experimental work in this direction, as well as little work from the perspective of empirical aesthetics. But this is changing. In recent work that I have done with colleagues, we have looked at how art experiences can influence well-being (Fekete, Specker, et al., in press; Trupp et al. 2022; Trupp et al. 2023) as well as pain and stress (Fekete et al., 2022; Fekete, Specker, Mikuni, Trupp, & Leder, in press). Trupp, et al. (2022, 2023) have shown a clear effect of art experiences on well-being; however, Trupp et al. (2022) showed similar effects of non-art (aesthetic food) engagement, making it unclear if there is anything “art specific” to the effects. Fekete et al. (2022) do use a clearly controlled design (with people looking at art, hearing music, or both, as well as looking at a gray screen); however, data collection is still ongoing. And, also for this work, it would need to be extended to compare to a non-art condition to assess the “art specificity” of the effects.

Similarly, we can also broaden this approach beyond art^2^ and ask how aesthetic experiences in general influence our daily life. This I am currently investigating with my team in my Austrian Science Fund (FWF) funded project (“The Function of Aesthetics in Our Lives,” P 35,140). In this project, we aim to understand how daily aesthetic experiences can play a role in our everyday life. And this last aspect is for me a crucial point, though an increase in experimental control and approaches are needed, we also need to focus on replicating findings in ecological settings in order to assess if eventual effects would also hold up outside the well-controlled lab environment.

In sum, I see developing into both these directions as important for future research, especially as calls for looking into well-being (Fancourt & Finn, 2019), flourishing (Cotter & Pawelski, 2021), and social epistemic outcomes of art (Sherman & Morrissey, 2017) are increasing.

## Methodologies of art/aesthetic experiences: theory, measurement, analysis

This discussion of experimental control brings me to my second point: methodological aspects of studying art or aesthetic experiences. This point, for me, centers around which statistical models we use to 1) formalize our theories, 2) develop our measurement instruments, and 3) analyze our data. I believe that in the future, we should take more care into ensuring a stronger connection between our theories and our methodological approaches.

With regard to analyzing data, I think we should move toward using (more complex) statistical models that reflect our theories better (see also Specker, Arató, & Leder, 2023). For example, most theoretical models (e.g. Leder et 2004; Locher, 2010; Pelowski et al. 2017; Tinio, 2013) conceptualize aesthetic experience as a process: assuming that both artwork as well as viewer characteristics are relevant. However, the statistical models we generally use to analyze our data – such as t-test, MANOVA, etc.—do not reflect this theoretical position. Rather than modeling an interaction, these models require averaging over stimuli using people as the degrees of freedom.^3^ This disconnect between theory and statistical model can be solved by using linear mixed models (LMM) – also called mixed effects, multilevel, or hierarchical models – that can incorporate the variance due to people, stimuli, and their interaction. Of course, my argument here is not to always blindly use an LMM, but rather that the statistical model employed should accurately reflect the underlying theory. Notably, this connection would be easier to establish if theoretical models were formalized (in terms of a statistical/psychometric model) which applies both to the developing of measurement instruments as well as theoretical models.

With regard to developing measurement instruments, though not necessarily consistently done, proposing – and testing! – a formalized psychometric model is required for thorough scale development as without it, the validity of the measurement instrument cannot be appropriately assessed. I will explain this point by discussing my development of the Vienna Art Interest Art Knowledge (VAIAK) scale (Specker, 2021; Specker et al., 2018; 2020; Specker, Arató, & Leder, 2023).

Importantly, a model can only be formulated based on a theoretical position. The formalization of the psychometric model is nothing different from putting your verbal theory into a numerical/mathematical form. For concerns of brevity, I refer the reader to Specker et al. (2020) for a detailed description of the theoretical background of the scale, and will only briefly summarize here. Our motivation to develop the VAIAK was to address two main problems in the field with respect to the study of “art expertise:” 1) a lack of conceptual clarity of what art expertise is, and 2) a lack of coherent measurement for assessing expertise. For the VAIAK we decided to limit the measurement to two constructs: interest and knowledge. Based on that these were most frequently studied (in the psychological literature). A fundamental theoretical idea here is that these are conceptualized as continuous: i.e. people can have more or less interest/knowledge, which is in contrast of much empirical literature that uses expertise as a dichotomy: you’re either an expert or you’re not – and empirically the two groups are then compared (see also Specker, 2021, for a more detailed discussion of this point). Both these aspects then fed into the psychometric model (specified in Specker, 2021) that specifies 2 latent variables (interest and knowledge) that predict the values of the items we developed for each corresponding subscale. The measurement of art interest and knowledge is thus also continuous (we advise a sumscore, see also Specker, Cotter et al., 2023).

As such, I believe that the scale can add theoretical clarity (i.e. what do we mean with “expertise”) or can at least spur on theoretical thinking and discussion. In addition, clarity in measurement makes it easier to understand empirical findings, i.e. are “expertise” effects due to interest or knowledge? Furthermore, the influence of interest/knowledge can be assessed without requiring an expert sample, which can lead to new insights. At least in my own work (Specker, Arató, & Leder, 2023; Specker, et al., 2023) I have found that art interest influences art perception – even in a group with *only* non-experts. Showing that these individual differences play a role beyond an expert/non-expert distinction.

Beyond this, the specification of a formal psychometric model also allows for testing this model and thus also potentially falsifying the underlying theory or giving concrete insight in how to change the scale. To briefly summarize, the model for the VAIAK has been corroborated by CFA (Specker, 2021). Nonetheless, the most recent work using an item-focused analysis using item-response theory (IRT) and qualitative analyses (Specker et al., 2023) still led to to an adaption of the original VAIAK scale to the VAIAK-R as 1 item clearly was not functioning properly.

This work does not stand alone, but is joined by e.g., the development of the Aesthetic Responsiveness Scale (AReA, Schlotz et al., 2021), the Art Affinity Index (AAI; Tschacher, Bergomi, & Tröndle, 2015) or the reassessment of existing scales such as the Visual Aesthetic Sensitivity Test (VAST; Myszkowski & Storme, 2017) or the Aesthetic Fluency Scale (AFS; Cotter et al., 2023; Cotter, Chen, Christensen, Kim, & Silvia, 2021). A list of questionnaires and tools for aesthetics, arts, and creativity research is even easily accessible on the OSF: https://osf.io/4s9p6/. Nonetheless, what I want to stress here is that simply developing a scale is not enough. Generally, continuous validation efforts – in any research area – are relatively scarce. It is time-intensive work with not much glory – the first development of the scale generally gets cited, continuous validation papers not so much – but it is essential to the quality of our measurement and thus, our scientific inferences. Finally, in order to engage in continuous validation, we need formalized models in order to be able to test if the underlying theory and conceptualization behind the measurement holds.

Which also brings me to my last point: formalizing our theoretical models more generally. The psychology of aesthetics/art is not lacking theoretical models. That said, box models (such as Leder, Belke, Oeberst, & Augustin, 2004; Pelowski et al., 2017) do not provide a (formalized) statistical model that can be tested. Instead, these broader integrative models provide a comprehensive overview of all psychological processes that may play a role. We need these broader perspectives, but we also need formalized theories to provide us with statistical models that can consequently be tested.

What does such a formalized model look like? In Specker et al. (2021), we provide such a model with the Aesthetic Effects Network (AEN). With the AEN, we aimed to provide an explicit account of one specific cognitive process involved in aesthetic experience. Specifically, the AEN represents an associative process where having one association (with an artwork) leads to the next association, generating an overall aesthetic experience.

This model was formalized in an interdisciplinary approach combining art history, psychology, and network science. Within art history, *aesthetic effect* is a key concept used to refer to associations (such as e.g. warm or sad) with artworks (Brinkmann et al., 2018). The idea that such an associative process is part of aesthetic experiences can actually be traced back to the founding father of empirical aesthetics Gustav Theodor Fechner (for an English translation of the relevant text see: Ortlieb, Kügel, & Carbon, 2020). Conceptualizing such an associative process as a network model is analogous to semantic networks such as word associations that have been widely described as a network (De Deyne & Storms, 2008; Steyvers & Tenenbaum, 2005).

In Specker, Fried, et al. (2023) we first outline the AEN based on these theoretical considerations as well as empirical data (*N* = 255) to generate a first version of the model. What is important to know here is that network models consist of nodes (in our case associations such as warm or sad) and edges (relationships between these nodes). The nodes that we included in our network were based mainly on art historical considerations on which associations have been considered theoretically (most) relevant. We then used our data to estimate the relationships between these nodes. We followed this up by a pre-registered study (*N* = 133) following calls in the literature to substantiate network theories by using experimental manipulation.

Specifically, we manipulated the brightness of the artworks. This manipulation was chosen based on psychological research showing that brightness of colors is generally associated with positivity (e.g., Lakens et al., 2013; Specker et al., 2018; Specker and Leder, 2018). As positivity was also a node in our network, we assumed that changing the brightness would also lead to a change in the positivity association. What we could show in this study was that by changing the value of one node (i.e. positivity) in the network (by experimental manipulation) we could predict changes in the other nodes. This finding supports the idea that the different nodes influence each other (i.e. an associative process from one association to the next).

Of course, the AEN only focuses on one cognitive process (aesthetic association) involved in aesthetic experience. However, the approach we took here can be used as a blueprint for future work to illustrate how to formulate and consequently test a formal model. Notably, this line of work does not stand alone. For example, Brielmann and Dayan (2022) have recently proposed a computational model of aesthetic value and computational predictive coding accounts have also been proposed for art (Sarasso et al., 2021; Van de Cruys & Wagemans, 2011).^4^ I would anticipate – and hope – that this formalization of theory would become more common in the future.

## Interdisciplinary research

The discussion of the AEN above also brings me to my final point: the importance of interdisciplinary research. If we want to understand art, including work from other disciplines that study art from different perspectives (such as art history, art theory, philosophy, as well as artistic research) can only help us enrich our understanding. For example, in both the AEN and the VAIAK projects discussed above, having an interdisciplinary team influenced our approach. In the case of the AEN, it led us to consider the potential importance of associative processes in aesthetic experience. Similarly, in the development of the VAIAK, it helped us develop our understanding of what can be considered “art knowledge” to then also develop items to test these different aspects of knowledge (in our case, knowledge about iconography or production methods).

Similarly, research done from the perspective of the humanities can be enriched by taking psychological work into account. For example, art theory normally assumes universality of aesthetic effects, but these assumptions are generally not empirically tested. In fact, when – again in an interdisciplinary approach – we did test this we could not find support for this assumption (Specker et al., 2018, 2020). As such, psychological methods can be useful in testing theoretical ideas from other disciplines. As also shown in the museum studies discussed above (Reitstätter et al., 2020; Specker et al., 2020). Both these studies went beyond looking at the museum context as a whole and studied specific aspects of the curation/display of art in museums. As a consequence, both showed that an interdisciplinary approach to museum research cannot only further the understanding of curatorial or museum studies but also the psychological understanding of how we engage with art.

Notably, interdisciplinary approaches are not limited to such art-related disciplines. Especially when aiming to propose statistical models, psychometrics, mathematics, network science, computer science, etc. can be important disciplines to consider as also shown by the discussion above. Thus, I can only stress the importance of such interdisciplinary collaboration for the future.

## Conclusion

Overall, I hope to have provided a brief overview of my work as well as some food for thought for the future. I aimed to show not just what I have done in the past, but also how this shaped the work that I am currently doing, the work that will (hopefully) soon hit the pages of scientific journals (i.e., the work under review), as well as the direction that I see my work developing in and that I believe should be pursued, not just by me but by many others. I chose this format in order to be able to show how I think the work I have done contributes to these developments and can, hopefully, also keep contributing in the future.

## Notes

1. As noted in almost all my recent work, I talk here about genuineness in terms of medium (i.e., a “real” artwork vs. a reproduction) rather than in terms of authenticity (a “real” Monet vs. a forgery).

2. And, of course, this approach can be broader than the dependent variables discussed here, for example work looking at a connection between art perception and creativity (Welke, Purton, & Vessel, 2021).

3. Notably, you can also do this the other way around which used to be common in psycholinguistics (Judd, Westfall, & Kenny, 2012) however, I’ve never seen this done in an aesthetics paper. Note that linear mixed models have since become the standard in psycholinguistics.

4. As far as I know for predictive coding no specific mathematical model has been proposed/tested for art/aesthetic experience, but as these build on other models of predictive coding for which computational details have been specified I do include them here.
